# The impact of particulate matter (PM2.5) on skin barrier revealed by transcriptome analysis: Focusing on cholesterol metabolism

**DOI:** 10.1016/j.toxrep.2019.11.014

**Published:** 2019-11-25

**Authors:** Zhengzheng Liao, Jing Nie, Peiwen Sun

**Affiliations:** Shanghai Chicmax Cosmetic Co., Ltd, Floor 38th, Global Harbor Building, 200036, Shanghai, China

**Keywords:** PM2.5, Transcriptome analysis, Cholesterol metabolism, Squalene, 3D-epidermis tissue model, Green tea extract

## Abstract

Accumulating evidence suggests that particulate matter (PM2.5), as a major air pollutant, imposes a certain degree of destruction and toxicity to the skin. It particularly impairs the structure and function of the epidermis. To study the impact of PM2.5 on the skin, transcriptome analysis was performed on PM2.5-exposed human primary keratinocytes. Functional annotation analysis demonstrates that PM2.5 significantly up-regulates cholesterol-metabolism-related genes. Via lipid extraction from PM2.5 treated three-dimensional epidermis tissue model (3D-ETM) and subsequent characterization via mass spectrometry, it was confirmed that PM2.5 significantly increases epidermal cholesterol levels *in vitro*. Conversely, the amount of squalene in 3D-ETM was significantly reduced by PM2.5. Interestingly, neither cholesterol nor squalene showed significant fluctuations in the green tea extract (GTE) treated epidermis tissue model under PM2.5 exposure. This study shows that PM2.5 may cause barrier disorders by increasing cholesterol synthesis, leading to transient accumulation of epidermal cholesterol and decrease of squalene. It was suggested that cholesterol and squalene, which are the key substances affecting skin barrier function, can be used as new biomarkers of skin damage induced by PM2.5. Moreover, it was demonstrated that GTE can reduce damage caused by PM2.5 exposure by not only anti-inflammatory and antioxidant mechanisms, but also by off-setting the disturbance to epidermal lipid homeostasis. This study demonstrates the strong potential of GTE as an active ingredient to be utilized in cosmetic products to effectively reduce the damage PM2.5 induces in skin.

## Highlights

• Transcriptome analysis revealed that PM2.5 significantly up-regulated cholesterol-metabolism-related genes.
• PM2.5 significantly increased the epidermal cholesterol level while reduced that of squalene in three-dimensional epidermis tissue model.
• Green tea extract was shown to reduce damage from PM2.5 exposure by off-setting the disturbance to epidermal lipid homeostasis.

## 1 Introduction

With the global industrialization, atmospheric pollutants have caused serious human health problems [1]. The World Health Organization (WHO) air quality guidelines identifies four major air pollutants, namely ground-level ozone, nitrogen dioxide, particulate matter and sulfur dioxide [2]. Among all these substances, PM2.5 is a small particulate matter, the aerodynamic diameter of which is less than 2.5 μm. As a key component of air pollution, PM2.5 imposes threats to the cardiovascular system, respiratory system and skin [3]. PM2.5 has a comparatively large specific surface area which can adsorb chemical pollutants and metal ions. It has been reported that prolonged exposure to particulate matters in the air can activate aryl hydrocarbon receptor (AhR), leading to extrinsic skin aging, wrinkle formation and changes in pigmentation [4]. Furthermore, skin diseases such as atopic dermatitis are induced and exacerbated by atmospheric pollutants [5].

The outer layer of the skin, also known as the stratum corneum (SC) is described as a “brick and mortar” structure. The “brick” refers to flattened, protein-enriched corneocytes, while the “mortar” refers to free fatty acids, ceramides, and cholesterol. The three lipid components are stacked in a highly ordered three-dimensional structure that “glues” the corneocytes together, forming a strong barrier [6]. The proper function of the skin barrier depends on the integrity of the SC, especially the composition of lipids in the SC. Di Nardo et al. found that the skin barrier was impaired in patients with atopic dermatitis, showing that ceramide levels in the SC were lowered, cholesterol increased, and the ceramide/cholesterol ratio reduced [7]. Although the lipid components have been studied as a measure of barrier integrity, little is known about the effect of air pollutants on the lipid composition in the SC. A number of clinical tests performed in 1999–2014 globally showed that residents living in areas with severe air pollution had high level of sebum oxidation and damaged SC [[8], [9], [10]]. Results from Mexico and Shanghai showed that people living in heavily polluted areas had higher sebum levels and a lower ratio of squalene/cholesterol, but no significant change in cholesterol levels [8,10].

As the key building blocks of the epidermis, keratinocyte-based *in vitro* models have been utilized to obtain mechanistic understanding of skin damage caused by external activators. Jin et al. studied the proteases stimulated by sulfur mustard in a normal human epidermis keratinocyte model [11]. Khalil et al. developed an assay system which utilized MTS, neutral red cytotoxicity and lactate dehydrogenase of HaCaT cells to characterize the cellular damage caused by UVB [12]. Keratinocyte models have also been applied to elucidate the mechanism of skin damage caused by chemical allergens, which can penetrate the skin barrier and cause allergic contact dermatitis. IL-1β and IL-18 were identified as key pro-inflammatory biomarkers of induction of allergic contact dermatitis in keratinocytes [13,14]. Furthermore, oxidative stress was shown to be the initial step in keratinocyte activation and thus is critical in allergic contact dermatitis [15]. In a HaCaT cell model, Potratz et al. showed that in addition to impacting the well-known cytochrome P450-dependent monooxygenases, polycyclic aromatic hydrocarbons (PAHs) also alter the lipid metabolite profile [16]. In this study, the goal was to understand the impact of PM2.5 on skin barrier by the transcriptome analysis of PM2.5-treated primary human epidermal keratinocytes (pHEK). Transcriptome analysis guided the focus to the changes in the expression of genes related to cholesterol metabolism. Then the impact of PM2.5 on cholesterol level was verified in a PM2.5 treated three-dimensional epidermis tissue model (3D-ETM).

Furthermore, the intervention of PM2.5-induced-skin damage by a plant-derived active ingredient was explored. *Camellia sinensis* is a traditional, economic plant and can be processed by different degrees of fermentation. Green tea is produced from fresh *C. sinensis* leaves which are carefully dried, to avoid the oxidation and polymerization of phenols. One of the major polyphenols found in green tea is epigallocatechin gallate (EGCG), which is a monomeric flavanol with strong anti-inflammatory and antioxidant effects. Studies have shown that green tea extracts can reduce UV-induced skin edema, erythema and protect DNA from UV-induced damage [17]. In this study, the effects of a green tea extract rich in polyphenols were tested at transcriptomic level as an intervention to the damage of PM2.5 to skin, and the changes of key lipid biomarkers in the 3D-ETM were verified by LC–MS.

## 2 Materials and methods

### 2.1 PM2.5 collection & analysis

PM2.5 sample was provided by the Institute of Earth Environment of the Chinese Academy of Sciences (Xi'an). PM2.5 from March to April 2009 at Xi'an High-tech Zone was collected at an airflow rate of 1200 L/min. The fine particles were trapped by a quartz fiber filter that was retrieved daily. Then the filter was sonicated in 40 mL of Milli-Q water for 15 min and repeated 3 times. After that, the suspension was dried using a vacuum freezer and stored at 4℃. Prior to cell treatment, PM2.5 was re-suspended in cell culture medium and sonicated for 30 min. Finally, the suspension was filtered with a glass fiber filter to remove the debris, making a final solution at 50 μg/mL of PM2.5. Methods of the chemical analysis of specific components have been reported previously in the literature [18]. Energy Dispersive X-Ray Fluorescence (ED-XRF) was used to determine the elemental composition of 12 elements (i.e., S, Ti, Cr, Mn, Fe, Ni, Cu, Zn, As, Br, Mo, Pb). To examine the amount of organic carbon (OC) and elemental carbon (EC) in the sample, an organic carbon analyzer was used.

### 2.2 Cell culture

pHEKs (PC2011, Biocell, Guangdong, China) were cultured in KcGrowth medium (PY1011, Biocell, Guangdong, China). pHEKs were cultured in a humidified 37 °C 5 % CO_2_ incubator. Sub-confluent keratinocytes were detached from the plate using EDTA-trypsin solution. Then the solution was centrifuged and diluted to 10^6^ cells/ml with culture medium. Cells were seeded in the plate at the density of 2 × 10^5^/well. After 24 h of incubation time, the culture medium was discarded and PM2.5 suspensions with or without green tea extract (GTE) were added to the culture plate. Three replicates were set for each experimental condition. The green tea extract (GTE) was a 20 % 1,3-butanediol aqueous solution with a dry weight of 0.2 %. The polyphenols, polysaccharides, amino acids and caffeine content of the GTE sample were 750 μg/ml, 2160 μg/ml, 252 μg/ml and 130 μg/ml respectively determined by standard analytical methods (See Supporting Information Table SI).

### 2.3 RNA isolation and sequencing

After keratinocytes were treated with PM2.5 or co-treated with GTE for 24 h, total RNA extraction was performed using the TRIzol Reagent (Invitrogen, USA) according to manufacturer’s instructions. RNA sample’s quality (degradation and contamination) was monitored on 1 % agarose gels. Spectrophotometer (NanoPhotometer, IMPLEN, USA) was used to check the purity. After measuring the concentration of RNA (with Qubit RNA Assay Kit, Qubit 2.0 Flurometer, Life Technologies, USA), the integrity of RNA was assessed (with RNA Nano 6000 Assay Kit, Bioanalyzer 2100 system, Agilent Technologies, USA). NEBNext Ultra RNA Library Prep Kit for Illumina (NEB, USA) was used for preparing the cDNA libraries. After that, the cDNA concentration was measured using AMPure XP system (Beckman Coulter, Beverly, USA), and were subjected to RNA-sequencing via Illumina HiSeq 4000 (Illumina, USA) using the Paired-End method. 150 bp paired-end reads were generated. Raw data of fastq format were filtered through in-house perl scripts to generate clean reads from which low-quality reads (Phred Quality Score ≤20) and reads containing adapter or poly-N were removed. TopHat v2.0.12 was used to align the clean reads to the reference genome, and HTSeq v0.6.1 was used to quantify the read numbers mapped to each gene. Differential expression analysis of different conditions was performed using the DEGseq R package (1.20.0). The P values were adjusted using the Benjamini & Hochberg method. The mapped data set could be found in “Supporting Information-mapped data of sequencing”.

### 2.4 Function annotation

Significantly up- and down-regulated genes were analyzed for gene ontology (GO) terms (biological processes, molecular function and cellular component) and KEGG Pathway using Metascape (http://metascape.org/gp/index.html#/main/step1).

### 2.5 Cell vitality assay

pHEKs were seeded at 1 × 10^4^ cells/well and cultured overnight. Then different levels of PM2.5 or PM2.5 + GTE solution were used to treat the cells respectively for 24 h. Afterwards, the medium was washed off with PBS. 20 μL MTT Reagent was added to each well and the plate was incubated at 37℃ for 4 h in the dark. Finally, the medium was discarded, followed by addition of 150 μL of DMSO. Absorbance at 490 nm was monitored by an ELISA Microplate Reader (BioTeK, USA) to quantify the cell vitality.

### 2.6 Cell morphology

Cell morphology was studied using an invert optical microscope (Olympus Corporation, Japan). Keratinocytes were incubated with PM2.5 or co-treated with GTE solution for 24 h. Morphological changes were observed. Two replicates were set for each experimental condition.

### 2.7 Extraction and characterization of cholesterol and squalene in PM2.5 treated epidermis tissue model

3D epidermis tissue model (Epikutis PM1011, Biocell, Guangdong, China) was cultured in EpiGrowth medium (PY1021, Biocell, Guangdong, China) at 37 °C 5 % CO_2_. After 4 days of tissue development, it was cultured in (1) the medium only, (2) the medium containing 50 μg/mL PM2.5, (3) the medium containing 50 μg/mL PM2.5 and 0.6 % GTE respectively for 2, 4, 6 days. The total volume of culture medium is 0.9 mL/well. The medium is replenished daily. Upon 3D-ETM sample collection, the medium was discarded and carefully wiped out from the surface of the epidermis. 3D-ETM were collected at different time points and stored at −20 °C, with 3 replicates at each condition. A spatula was used to carefully detach the 3D-ETM from the sides of the well. Then the sample was digested with 100 μL of proteinase K (Ambion) at 55 °C for 30 min. Samples cultured at the same condition were combined and sonicated in organic solvent (chloroform: methanol = 2:1) under ice-water bath to extract the lipid components. Afterwards, the samples were dried under nitrogen and stored at −80 °C prior to LC–MS analysis.

For LC–MS, liquid chromatography was equipped with a C18, 1.8 μM, 100 × 2.1 mm column. The lipid sample was re-dissolved in the mobile phase and the volume loaded was 1 μL. Two mobile phases used were: A. acetonitrile: isopropanol = 1:9 (v/v), 0.1 % formic acid; and B. water: acetonitrile = 4:6 (v/v), 0.1 % formic acid. The mobile phases were set as follows: flow rate: 0.5 ml/min, a linear gradient of B from 99 % to 50 % for 7 min, a linear gradient of B from 50 % to 1 % for 3 min, an isocratic elution of B at 1 % for 3 min, and finally an isocratic elution of B at 99 % from 3 min for column equilibrium. Parameters for Orbitrap MS was as follows: positive mode, spray voltage = 4000 V, gas pressure 1, = 30 psi, gas pressure 2 = 10 psi; scan range = 150–1000 *m/z*. The analysis was performed using Progenesis QI.

## 3 Results

### 3.1 Characterization of PM2.5 sample

Due to the different composition of PM2.5 based on region, chemical analysis of the PM2.5 samples collected from Xi'an, China was performed (Table 1). PM2.5 from Xi’an contains complex components such as metal ions, organic and inorganic substances. Several studies focus on the analysis of their chemical constituents [19] and the adverse effects on human health [20]. Elemental composition analysis (Table 1) shows that S, Zn, and Fe are the top three components of the PM2.5 used in the experiment. Zn and Fe are among the most abundant crustal elements, indicating that dust emission is an important source of this sample. Meanwhile, high concentration of S, NO_3_^−^ and SO_4_^2-^ indicates that coal combustion emission is another important source. Sulfate and nitrate are produced from the oxidation of SO_2_ and NO_X_, which is considered to be mostly produced by coal combustion in China. Furthermore, it has been reported that OC/EC of coal combustion emissions is 2.5–10.5 [21]. In this experiment, OC/EC is 5.25, which again shows the major contribution of coal combustion emission. Hence, these results suggest that the PM2.5 sample is originated from coal-combustion and crustal dust.

**Table 1:** Chemical analysis of PM2.5 sample collected in Xi'an China.

Elemental species(μg/m^3^)											
S	Ti	Cr	Mn	Fe	Ni	Zn	As	Br	Mo	Cd	Pb
3.4	0.12	0.01	0.13	1.42	0	2.28	0.02	0.06	0.05	0.02	0.29
Ionic species(μg/m^3^)											
		K^+^	Na^+^	NH_4_^+^	NO_3_^−^	SO_4_^2−^	Mg^2+^	Ca^2+^	Cl^−^	F^−^	
		1.14	1.5	4.32	9.32	13.3	0.18	2.24	3.83	0.15	
Carbonaceous components(μg/m^3^)											
Total carbon		Organic carbon			Elemental carbon			water-soluble organic carbon			
26.7		22.43			4.27			7.62			

### 3.2 Effect of PM2.5 on keratinocyte viability

**Fig. 1:**
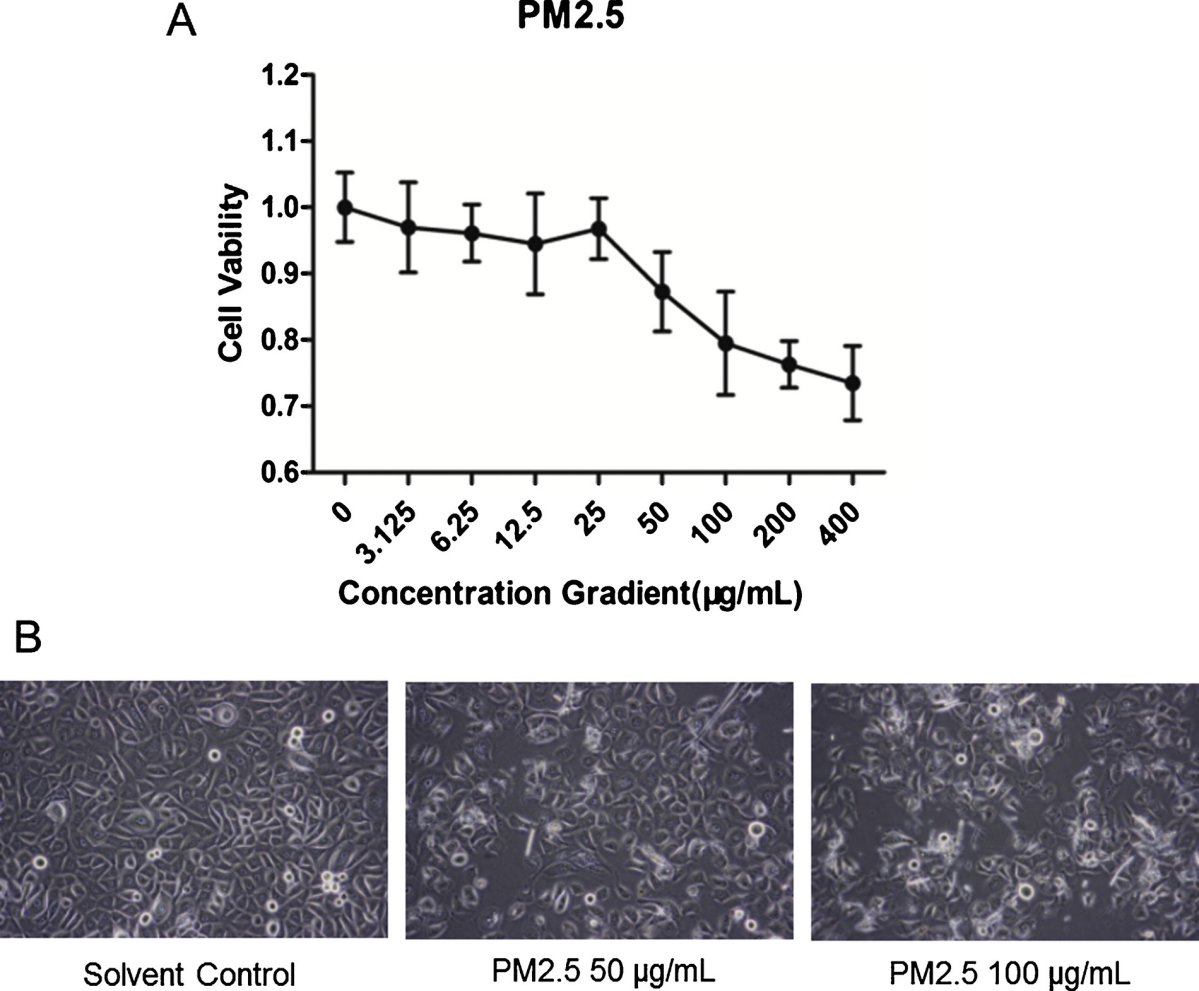
The viability (A) and morphology (B) of keratinocytes treated with different concentrations of PM2.5.

The toxicity of PM2.5 to pHEKs was detected by MTT assay and morphological study. After treatment with PM2.5 C(PM2.5) >50 μg/mL for 24 h, a progressive decrease of cell viability was shown in a dose-dependent manner (Fig. 1). In terms of cell morphology, when the PM2.5 dosage was increased to 50 μg/mL, some cell debris starts to appear. Cells become slightly pyknotic, but there isn’t any significant change in the number of adherent cells. When the concentration of PM2.5 was further increased, there are many adherent cells shrinking, rounding, and detaching and the number of adherent cells was significantly reduced. Therefore, the maximum dosage of PM2.5 selected for subsequent experiments was 50 μg/mL, which didn’t significantly affect the viability of the cells.

### 3.3 Effect of PM2.5 on mRNA expression in keratinocytes

**Fig. 2:**
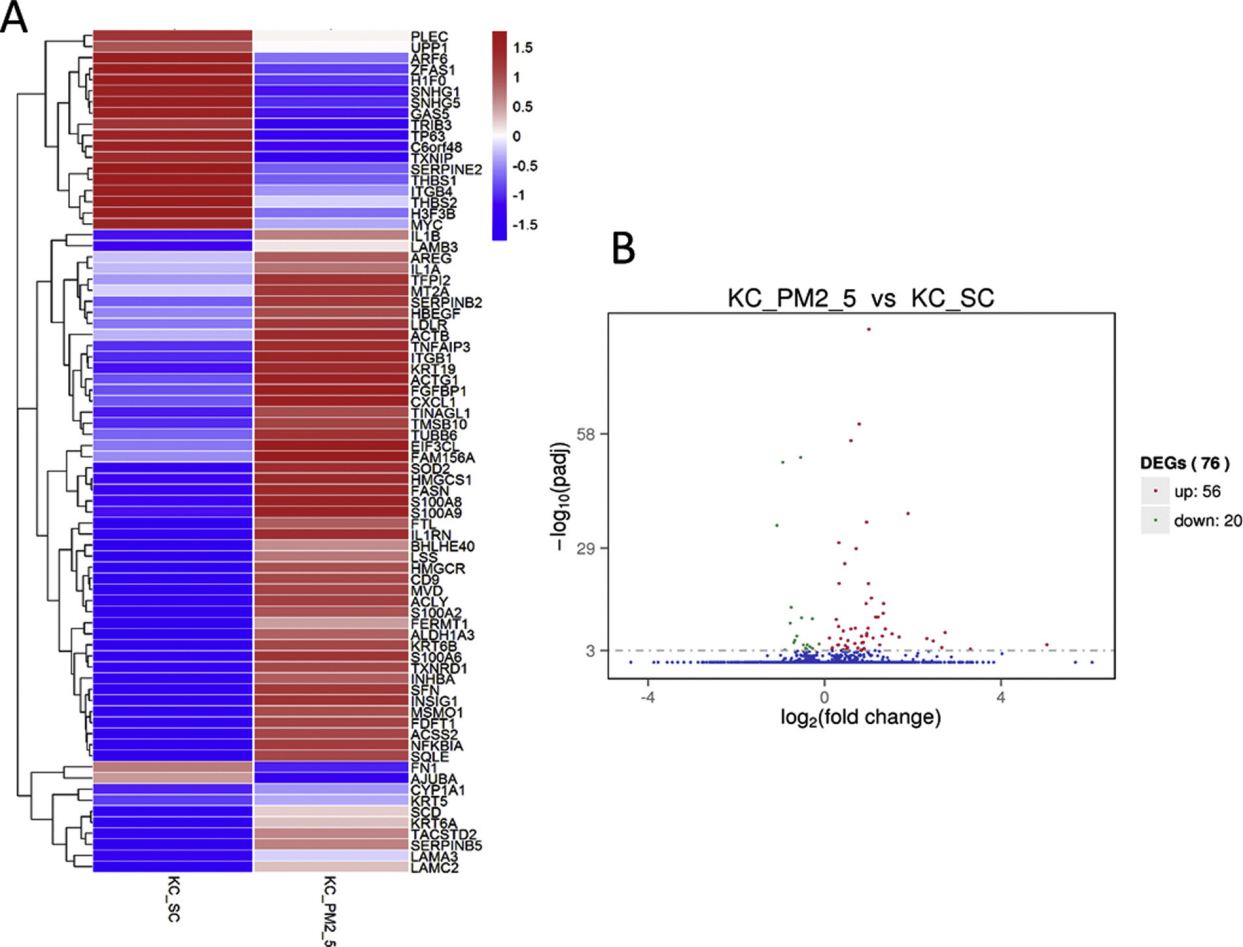
Gene expression profiles of PM2.5-treated group (KC_PM2.5) vs. control group (KC_SC). (A) The heat map showing the gene expression levels of keratinocytes under the treatment of PM2.5 versus the control group. The color indicate the relative expression level of the gene. Only genes with padj< 0.001 were shown in the heat map. padj refers to the multiple testing corrected p values. (B) The volcano plot. The horizontal axis represents fold change; while the vertical axis represents statistically significant difference. Red dots: significantly up-regulated genes, green dots: significantly down-regulated genes.

Using the Illumina sequencing technology, we performed comprehensive gene expression profiling and investigated the changes induced by PM2.5 in human keratinocytes. The rRNA sequence and low-expression sequences were filtered. Significant differences in expression of the PM2.5-treated group in comparison with the control group was defined as P_adj_<0.001. Among them, 56 genes were significantly up-regulated while 20 genes were significantly down-regulated. The heat map and volcano plots were shown in Fig. 2.

The top 30 significantly up-regulated genes were ranked according to the fold change in Table 2. Among them, chemokine (C-X-C motif) ligand 1 (CXCL1) and cytochrome P450 family 1 subfamily A member 1 (CYP1A1) were most significantly up-regulated by PM2.5. CXCL1 is a keratinocyte-derived chemokine involved in inflammatory response [22]. CXCL1 participates in the IL-17 signaling pathway, which is closely related to psoriasis [23]. In addition, the mRNA of S100A8, S100A9, TNFAIP3, IL-1a, and IL-1b, which are closely related to inflammation, were also significantly increased consistent with previous reports [24]. It was further verified that PM2.5 can induce a significant inflammatory response in keratinocytes. Moreover, CYP1A1, a member of the cytochrome P450 family, was highly up-regulated. CYP1A1 is responsible for the metabolism of toxic compounds such as polycyclic aromatic hydrocarbons (PAHs) produced by the combustion of tobacco [25]. It is reported that CYP1A1 also responds to the stimulation of PM2.5 [24]. It is also worth noting that the gene expression level of superoxide dismutase 2 (SOD2), which is involved in cellular oxidative stress, is significantly increased by PM2.5 stimulation [26]. Lastly, inhibin beta-related genes A (INHBA), a gene involved in the regulation of skin extracellular matrix differentiation and apoptotic homeostasis [27], is also up-regulated after the treatment of PM2.5.

**Table 2:** Top 30 significantly up-regulated genes in the keratinocytes treated with PM2.5.

Associated Gene Name	log2.Fold change.	pvalue	padjue	Description
CXCL1	3.3047	1.37E-06	0.00045496	chemokine (C-X-C motif) ligand 1 (melanoma growth stimulating activity, alpha)
CYP1A1	2.7319	4.01E-11	2.83E-08	cytochrome P450 family 1 subfamily A member 1
S100A8	2.6565	5.16E-07	0.0001883	S100 calcium binding protein A8
EIF3CL	2.4628	7.74E-09	3.76E-06	eukaryotic translation initiation factor 3 subunit C-like
S100A9	2.319	1.79E-09	9.31E-07	S100 calcium binding protein A9
HMGCS1	1.8909	4.51E-42	1.64E-38	3-hydroxy-3-methylglutaryl-CoA synthase 1
IL1RN	1.689	6.44E-10	3.71E-07	interleukin 1 receptor antagonist
INSIG1	1.5304	9.01E-11	5.98E-08	insulin induced gene 1
KRT6B	1.3754	4.66E-12	3.51E-09	keratin 6B, type II
TNFAIP3	1.3361	8.01E-19	1.17E-15	TNF alpha induced protein 3
SERPINB2	1.3277	2.99E-16	3.63E-13	serpin peptidase inhibitor, clade B (ovalbumin), member 2
SOD2	1.3093	3.74E-10	2.27E-07	superoxide dismutase 2, mitochondrial
NFKBIA	1.2489	9.67E-08	3.92E-05	nuclear factor of kappa light polypeptide gene enhancer in B-cells inhibitor, alpha
TXNRD1	1.2116	2.89E-15	3.28E-12	thioredoxin reductase 1
TACSTD2	1.1573	3.00E-15	3.28E-12	tumor-associated calcium signal transducer 2
HBEGF	1.1016	1.27E-09	6.94E-07	heparin-binding EGF-like growth factor
MSMO1	1.0632	3.23E-20	5.04E-17	methylsterol monooxygenase 1
FASN	1.0064	1.30E-89	2.83E-85	fatty acid synthase
ACLY	0.99489	6.19E-24	1.04E-20	ATP citrate lyase
ALDH1A3	0.98715	2.55E-12	2.15E-09	aldehyde dehydrogenase 1 family member A3
SQLE	0.9585	6.93E-11	4.74E-08	squalene epoxidase
ACSS2	0.95247	2.72E-10	1.75E-07	acyl-CoA synthetase short-chain family member 2
FGFBP1	0.95138	7.90E-40	2.47E-36	fibroblast growth factor binding protein 1
FTL	0.93974	8.68E-19	1.19E-15	ferritin, light polypeptide
BHLHE40	0.89669	9.37E-07	0.00031543	basic helix-loop-helix family member e40
KRT19	0.87714	1.83E-08	8.70E-06	keratin 19, type I
HMGCR	0.8467	2.49E-08	1.13E-05	3-hydroxy-3-methylglutaryl-CoA reductase
IL1B	0.83678	2.44E-06	0.00073037	interleukin 1 beta
TFPI2	0.8355	3.93E-10	2.33E-07	tissue factor pathway inhibitor 2
INHBA	0.82203	3.44E-06	0.00099125	inhibin beta A

### 3.4 Functional annotation and pathway analysis of keratinocytes treated with PM2.5

**Fig. 3:**
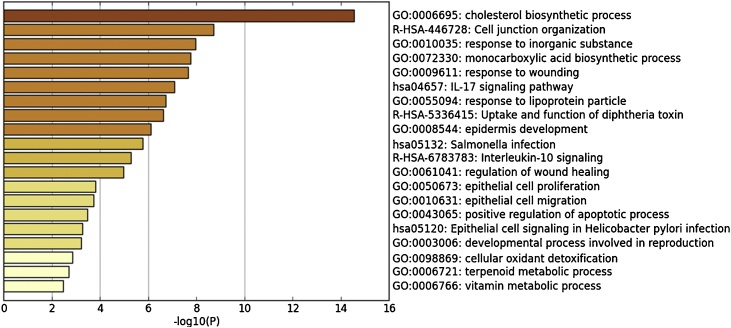
Top 20 GO-terms and pathways of up-regulated genes stimulated by PM2.5.

In order to further explore the unique signaling pathways and protein networks that respond to the stimulation of PM2.5, Metascape was used as the bioinformatic source to analyze the gene expression data set. Through the analysis, information such as the biochemical pathways involved, subcellular localization of the end-products from the gene transcription, translation as well as the relevance to certain diseases can be obtained. The data set used for this analysis was genes that are significantly up-regulated after PM2.5 treatment as compared to control (p_adj_<0.001). The results are shown in Fig. 3.

The annotation results are ranked according to the degree of enrichment in descending order. The top 20 GO terms and pathways are shown in the figure above (Fig. 3); the most highly enriched GO term is cholesterol biosynthetic process (GO: GO:0006695) suggesting that PM2.5 may influence cholesterol biosynthesis in the skin. Moreover, the response to lipoprotein particle (GO:0055094) was also among the top enriched terms, pointing to the close relationship between PM2.5 and cholesterol metabolism in cells.

In addition, inflammation-related pathways such as IL-17 signaling pathway (hsa04657), Interleukin-10 signaling (R-HSA-6783783) are also highly enriched. Meanwhile, response to wounding (GO:0009611), regulation of wound healing (GO:0061041), which are among the top 20, are also involved in the skin inflammation response. The identification of positive regulation of apoptotic process (GO:0043065) and the cellular oxidant detoxification (GO:0098869) pathways revealed that PM2.5 may induce cell apoptosis and intracellular oxidative clearance as a protective measure.

In summary, through functional annotation analysis, cholesterol biosynthesis, inflammation, oxidative stress, and the closely related apoptotic pathways, are the most activated pathways in PM2.5 treated keratinocytes.

### 3.5 GO analysis predicts abnormal cholesterol metabolism in PM2.5 treated keratinocytes

Lipids are the main components of the cell membrane, an important part of the SC and crucial for the normal function of the skin barrier. Studies have shown that external stimulation such as acetone increases epidermal lipid synthesis [28]. “Cholesterol biosynthetic process” and “Respond to lipoprotein particle” stands out among the most enriched terms from GO analysis. Hence, the related genes were further analyzed in detail.

**Fig. 4:**
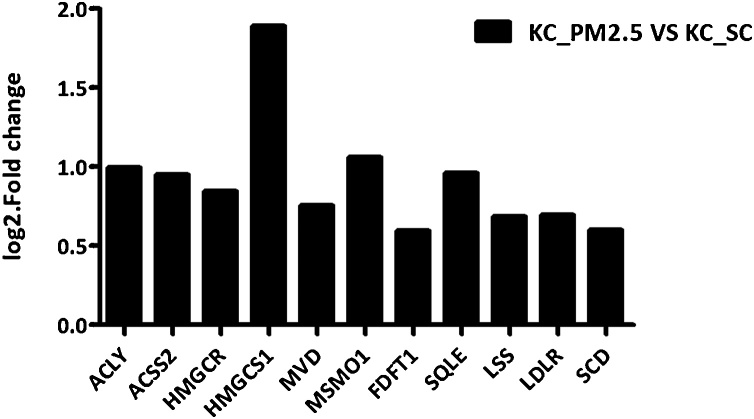
Significantly up-regulated genes involved in cholesterol metabolism.

The genes associated with the cholesterol metabolism are listed in Table 3, and its expression level in comparison with the control is plotted in Fig. 4. A clear map of the cholesterol biosynthesis pathway can be found in the article published by Mazein et al. [29]. The cholesterol synthesis process is divided into three phases. First, acetyl-CoA forms mevalonate; secondly, two mevalonic acids are condensed into one isoprene, followed by the formation of squalene; and third, squalene is converted into cholesterol. The whole cholesterol synthesis process involves at least 23 genes. Both cholesterol and fatty acid synthesis begin with a common precursor, acetyl-CoA, which is derived from the catabolism of sugars, proteins, and lipids. Among the genes induced by PM2.5 (Table 3), ACLY, ACSS2 is involved in the production of acetyl-CoA. HMGCS1, HMGCR participate in the first stage of cholesterol synthesis. MVD, FDFT1 participate in the second stage of cholesterol synthesis [29]. LSS, and SQLE participate in the synthesis of lanosterol by squalene in the third stage of cholesterol synthesis. LDLR encodes the protein receptor that binds to the carriers of cholesterol-low density lipoproteins (LDLs). Hence, it is key for the uptake of cholesterol into cells. As a result, it is deduced that the up-regulation of these genes under the treatment of PM2.5 may lead to increased cholesterol synthesis.

**Table 3:** Cholesterol metabolism related genes.

Gene Symbol	log2.Fold_change.	pvalue	padjue	Description	Role in cholesterol metabolism
ACLY	0.99489	6.19E-24	1.04E-20	ATP citrate lyase	Acetyl-Co synthesis
ACSS2	0.95247	2.72E-10	1.75E-07	acyl-CoA synthetase short-chain family member 2	
HMGCR	0.8467	2.49E-08	1.13E-05	3-hydroxy-3-methylglutaryl-CoA reductase	Cholesterol synthesis step1
HMGCS1	1.8909	4.51E-42	1.64E-38	3-hydroxy-3-methylglutaryl-CoA synthase 1	
MVD	0.75521	6.24E-07	2.22E-04	mevalonate diphosphate decarboxylase	Cholesterol synthesis step2
MSMO1	1.0632	3.23E-20	5.04E-17	methylsterol monooxygenase 1	
FDFT1	0.59669	4.65E-12	3.51E-09	farnesyl-diphosphate farnesyltransferase 1	
SQLE	0.9585	6.93E-11	4.74E-08	squalene epoxidase	Cholesterol synthesis step3
LSS	0.68795	3.73E-08	1.66E-05	lanosterol synthase	
LDLR	0.69696	2.72E-12	2.20E-09	low density lipoprotein receptor	Cholesterol transfer
SCD	0.59881	7.25E-61	5.29E-57	stearoyl-CoA desaturase	Fatty acid synthesis
FASN	1.0064	1.30E-89	2.83E-85	fatty acid synthase	
INSIG1	1.5304	9.01E-11	5.98E-08	insulin induced gene 1

### 3.6 The effect of GTE on PM2.5 stimulated keratinocytes

To explore the effect of green tea extract on PM2.5-stimulated cells, we tested the concentration of green tea extract (GTE) in keratinocytes using MTT assay and morphological study (Supporting Information Fig. S1). C_(GTE)_ = 0.6 % was selected because under such concentration the cell viability is not affected.

**Fig. 5:**
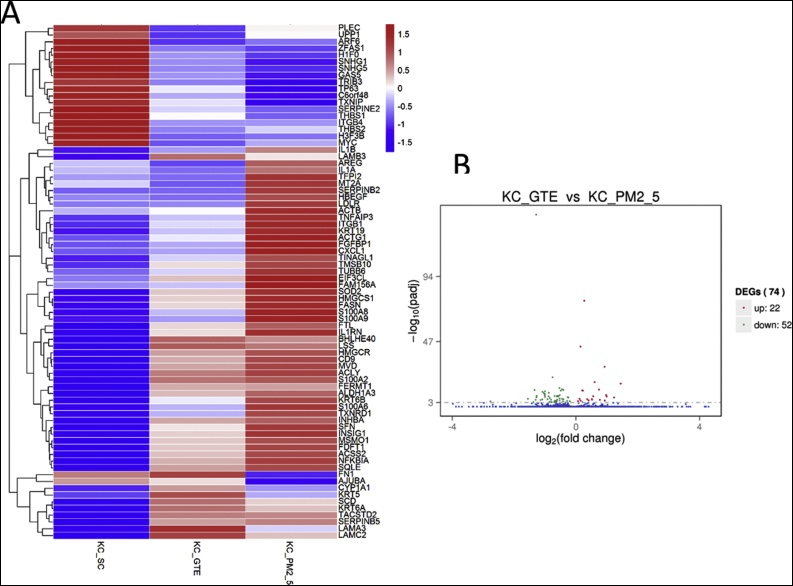
Gene expression profiles of control (KC_SC), GTE + PM2.5 co-treated (KC_GTE), and PM2.5 treated (KC_PM2.5) samples (A) The heat map showing the gene expression levels of keratinocytes of control, under the treatment of GTE and PM2.5, as well as under the treatment of PM2.5 only. Only genes with padj< 0.001 were shown in the heat map. (B) The volcano plot.

Cells exposed to PM2.5 alone and with GTE were studied by RNA-Seq. The heat map is shown in Fig. 5A, demonstrating that GTE can offset the changes in expression level of genes previously influenced by PM2.5. In the volcano plot (Fig. 5B), it is shown that in the group co-treated with PM2.5 and GTE, 22 genes are significantly up-regulated while 52 genes are significantly down-regulated compared to PM2.5 treatment only. Furthermore, results demonstrate that GTE acts mainly by counterbalancing the influence of PM2.5 on keratinocytes.

**Fig. 6:**
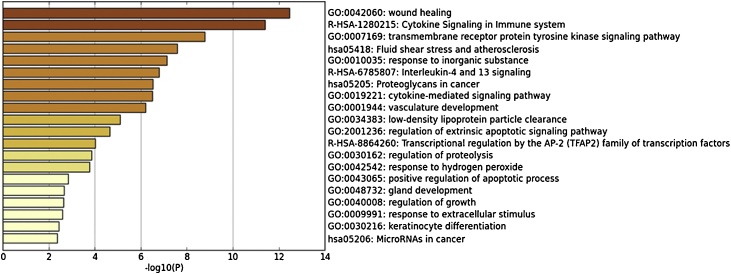
Top 20 GO-terms and pathways of significantly down-regulated genes in PM2.5 and GTE co-treated group.

Similarly, Metascape was used to analyze the significantly down-regulated genes for the PM2.5 and GTE co-treated group in comparison with the group with PM2.5 treatment only (P < 0.001) (Fig. 6). In addition, the differentially expressed genes is listed in Supporting Information Table SⅡ.

The results showed that the Cytokine Signaling in Immune system (R-HSA-1280215) and wound healing (GO:0042060), which are closely related to the inflammatory response, were the most enriched terms that were down-regulated after GTE treatment. In addition, there are four other terms involved in the inflammatory response, namely: cytokine-mediated signaling pathway (GO:0019221), Interleukin-4 and 13 signaling (R-HSA-6785807), response to extracellular stimulus (GO:0009991). Therefore, it can be inferred that GTE primarily relieves PM2.5-induced inflammatory responses. In addition, the GO term: response to hydrogen peroxide (GO:0042542) indicating that GTE also acts an antioxidant, which relieves the oxidative stress induced by PM2.5. Regarding the synthetic pathway of cholesterol, although it is not shown in GO analysis, green tea extract significantly down-regulates the expression of HMGCS1 and LDLR genes (Supporting Information Table SⅡ), which are involved in cholesterol metabolism. The transcriptome analysis showed that the protective effect of GTE on cells may also be reflected in the restoration of the balance of cholesterol metabolism.

### 3.7 PM2.5 affects cholesterol and squalene content in epidermis tissue model

**Fig. 7:**
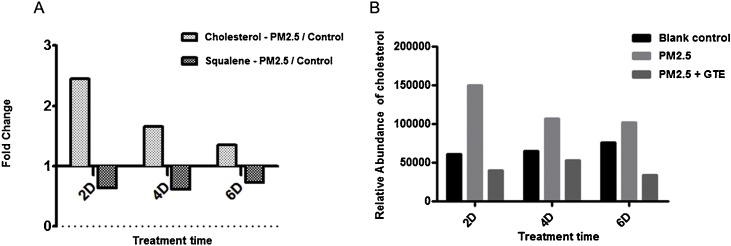
(A) Fold change in Cholesterol and Squalene level characterized by LC–MS in 3D-ETM. PM2.5/Control: PM2.5-treated 3D-ETM/control 3D-ETM (B) Cholesterol level quantified in epidermis tissue models by LC–MS.

Transcriptomic studies suggest that PM2.5 could disturb cholesterol homeostasis. To verify whether the final cholesterol level in the skin has changed, 3D *in vitro* skin model was treated with PM2.5 or co-treated with GTE + PM2.5. The cholesterol and squalene levels were characterized at different treatment times at Day 2, 4 and 6. Result are shown in Fig. 7. Under the treatment of PM2.5, cholesterol levels were much higher than that of the control group (Fig. 7A). The increase of cholesterol in the skin was the most significant on Day 2, which was about 2.5 times more as compared to the control group. Then the cholesterol level gradually decreased over day 4 to day 6. In contrast, squalene, which is a precursor of cholesterol, was significantly reduced after treatment of PM2.5. This suggests that more squalene is used for the synthesis of cholesterol under PM2.5 stimulation. Addition of GTE inhibits the increase of cholesterol induced by PM2.5, leading to equal levels of cholesterol as compared to the control group (Fig. 7B).

## 4 Discussion

In this study, the PM2.5 sample, which originates from coal combustion and crustal dust, is shown to up-regulate inflammatory, oxidative-stress-response, and AhR pathways in human primary keratinocytes. More importantly, gene cluster analysis reveals that besides the above-mentioned biological processes, PM2.5 significantly up-regulates many genes involved in cholesterol metabolism, such as ACLY、ACSS、HMGCS1、HMGCR、MVD、FDFT1、SQLE、LSS、LDLR, etc. Most of these genes encode enzymes that play important roles in various stages of acetyl-CoA de novo synthesis of cholesterol. LDLR mainly participates in the transfer of cholesterol, mediating the endocytosis of cholesterol-rich LDL to absorb and utilize lipoprotein-carrying cholesterol [30]. It is further confirmed that the actual cholesterol levels in 3D skin model is significantly increased under the influence of PM2.5.

It has been reported that the cholesterol level is an important indicator of the skin barrier function: in the epidermis of atopic dermatitis patients, the cholesterol level is significantly higher than that in healthy subjects [7]. Previous studies have also shown that HMG-CoA reductase (HMGCR) and low-density lipoprotein receptor (LDLR) are significantly increased at the transcriptional and translational levels under both acute barrier destruction caused by acetone or tape stripping and chronic barrier destruction caused by fatty acid deficiency [31]. It is suggested that barrier disruption induces lipid synthesis and intercellular trafficking to promote barrier repair. In this study, the transcription of HMG-CoA reductase in keratinocytes was up-regulated after PM2.5 treatment, and the cholesterol content in the 3D skin model increased significantly on the second day of PM2.5 treatment, then gradually decreased on day 4 and day 6. Since cholesterol is an important component of cell membranes, when damage occurs, the skin accelerates the rate of cell proliferation producing more keratinocytes to compensate for lesion sites [32]. This may explain why the cholesterol level gradually decreases on day 4, 6 as compared to day 2 after PM2.5 treatment. In summary, PM2.5 could lead to up-regulation of cholesterol synthesis, disrupting cholesterol homeostasis. Under PM2.5 exposure, a large amount of cholesterol is synthesized to supplement keratinocytes, intercellular lipids and repair the damaged SC.

A compensatory increase in cholesterol level may further affect skin physiology. Cholesterol is also an important component of sebum. Study has shown that an increase in sebum has a negative effect on the epidermal barrier [33]. Moreover, the accumulation of cholesterol can cause the up-regulation of inflammatory responses, mainly by triggering the downstream signaling pathway of IL-1 [34].

Another important question addressed in this study is the effect of green tea extract in preventing the damage caused by PM2.5. Although EGCG from green tea is known for its significant antioxidant effect [35], the specific effect of EGCG or green tea extract against the particulate matters has not been explored. Our experiment showed that when the appropriate concentration of green tea extract was added to PM2.5-treated keratinocytes, many genes up-regulated by PM2.5 returned to normal level. The gene cluster analysis indicates that the related genes were mainly concentrated in the inflammatory response and oxidative-stress pathway. In terms of cholesterol-metabolism-related genes, green tea extract significantly down-regulated the expression of HMGCS1 and LDLR genes. Finally, it was demonstrated that the addition of green tea to PM2.5-exposed skin tissue could significantly reduce the cholesterol level in 3D skin model.

## 5 Conclusion

Through transcriptome study of PM2.5-exposed pHEK, the effect of PM2.5 on the cholesterol metabolism was discovered, demonstrating the increase of cholesterol and decrease of squalene in a PM2.5-exposed 3D skin model. Moreover, the data demonstrates that green tea extract (GTE) effectively inhibits the damage induced by PM2.5, including reversing PM2.5 stimulated cholesterol synthesis. This suggests that GTE has protective effects on skin damage caused by fine-particulate matter.

## Declaration of Competing Interest

The authors declare no conflict of interest.
