# Supplementary material for: The impact of particulate matter (PM2.5) on skin barrier revealed by transcriptome analysis: Focusing on cholesterol metabolism
Source: Toxicol Rep. 2019 Nov 25;7:1–9. doi: 10.1016/j.toxrep.2019.11.014 (PMC6906712; doi:10.1016/j.toxrep.2019.11.014)
Supplement: Supplementary file 1 [file mmc1.doc]

**Supporting Information**

**The impact of PM2.5 on skin barrier revealed by transcriptome analysis: focusing on cholesterol metabolism.**

**Figure S1. The viability (A) and morphology (B) of keratinocytes treated with different concentrations of green tea extract (GTE).**

**
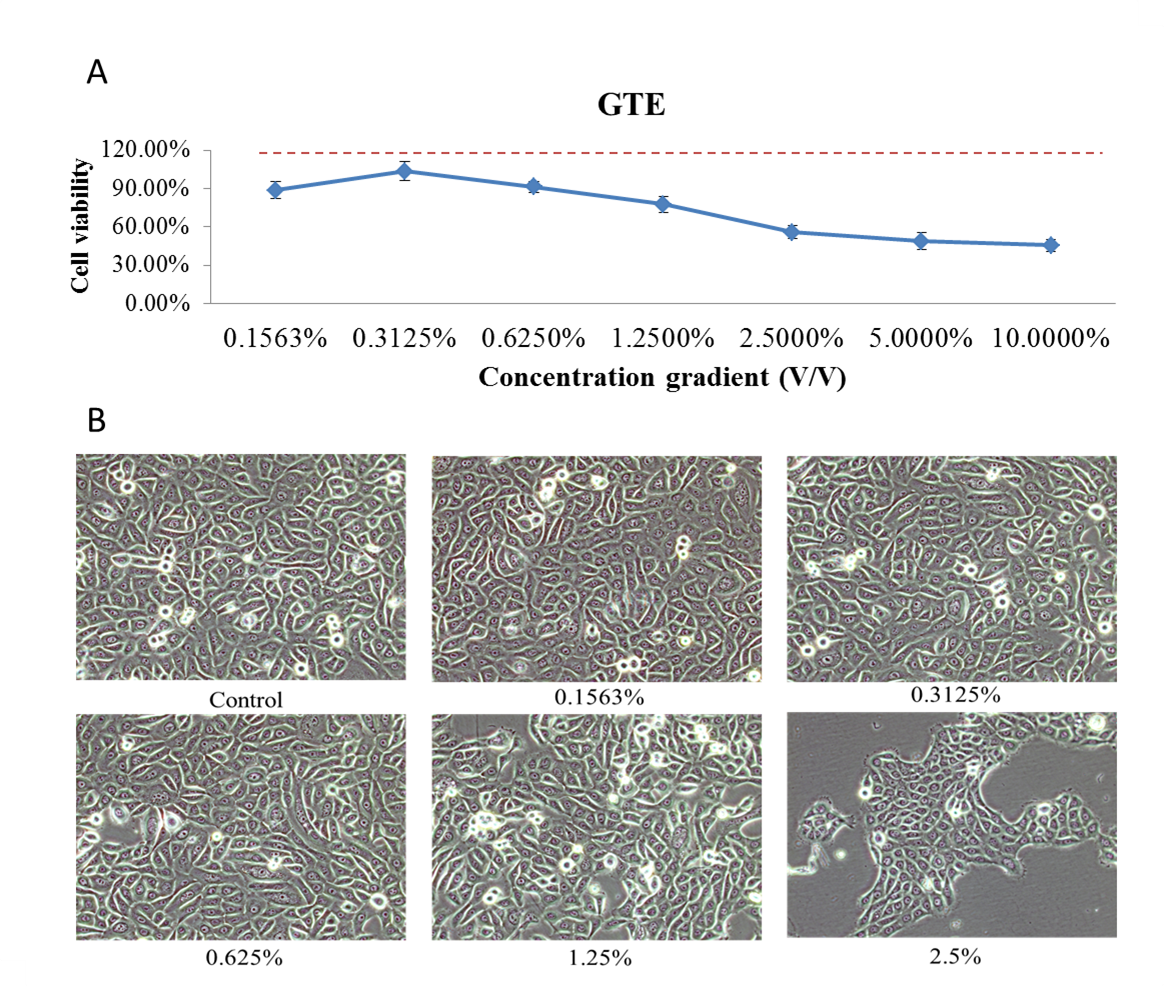
**

**Table SⅠ.Chemical components of green tea extract determined by standard analytical methods**

| **Components** | **Conc. (μg/mL)** | **Analytical Method** |
| --- | --- | --- |
| polyphenols | 750 | Folin & Ciocalteu's method |
| polysaccharides | 2160 | Phenol-sulfuric acid method |
| amino acids | 252 | Ninhydrin colorimetric method |
| caffeine | 130 | High-performance liquid chromatography |

**Table SⅡ. Genes up-regulated in PM2.5 treated group vs. control group, while down-regulated in PM2.5+GTE treated group vs. PM2.5 treated group.**

| Associated Gene Name | Description | log2FoldChange(KC_PM2_5vsKC_SC) | log2FoldChange(KC_GTEvsKC_PM2_5) | pval(KC_PM2_5vsKC_SC) | pval(KC_GTEvsKC_PM2_5) |
| --- | --- | --- | --- | --- | --- |
| TXNRD1 | thioredoxin reductase 1 | 1.2116 | -0.59674 | 2.890E-15 | 4.800E-05 |
| TUBB6 | tubulin beta 6 class V | 0.52809 | -0.3987 | 6.010E-09 | 3.480E-05 |
| TUBB4B | tubulin beta 4B class IVb | 0.564 | -0.43213 | 4.190E-06 | 8.043E-04 |
| TNFAIP3 | TNF alpha induced protein 3 | 1.3361 | -0.80529 | 8.010E-19 | 1.910E-08 |
| TFPI2 | tissue factor pathway inhibitor 2 | 0.8355 | -1.0404 | 3.930E-10 | 1.290E-13 |
| SERPINB2 | serpin family B member 2 | 1.3277 | -1.3377 | 2.990E-16 | 5.530E-16 |
| SERPINB1 | serpin family B member 1 | 0.58058 | -0.5925 | 7.598E-04 | 9.901E-04 |
| S100A9 | S100 calcium binding protein A9 | 2.319 | -1.3048 | 1.790E-09 | 8.130E-05 |
| PTGS2 | prostaglandin-endoperoxide synthase 2 | 0.46721 | -0.65546 | 6.474E-04 | 5.750E-06 |
| PLAU | plasminogen activator, urokinase | 0.37333 | -0.56674 | 3.780E-06 | 2.890E-11 |
| MT2A | metallothionein 2A | 0.78451 | -1.2851 | 3.010E-65 | 9.710E-144 |
| MMP1 | matrix metallopeptidase 1 | 1.421 | -1.9327 | 4.662E-04 | 1.450E-05 |
| MALL | mal, T-cell differentiation protein like | 1.202 | -1.426 | 1.416E-04 | 1.730E-05 |
| LDLR | low density lipoprotein receptor | 0.69696 | -0.68895 | 2.720E-12 | 2.330E-11 |
| KRT19 | keratin 19 | 0.87714 | -0.54891 | 1.830E-08 | 4.608E-04 |
| ITGB1 | integrin subunit beta 1 | 0.30003 | -0.22488 | 1.020E-07 | 5.788E-04 |
| IL1A | interleukin 1 alpha | 0.42438 | -0.62145 | 2.050E-06 | 4.500E-11 |
| IER3 | immediate early response 3 | 0.74513 | -0.57801 | 8.230E-06 | 7.267E-04 |
| HSPA8 | heat shock protein family A (Hsp70) member 8 | 0.47634 | -0.64266 | 4.460E-05 | 1.730E-07 |
| HSP90AA1 | heat shock protein 90 alpha family class A member 1 | 0.28074 | -0.52262 | 1.050E-04 | 5.610E-12 |
| HMGCS1 | 3-hydroxy-3-methylglutaryl-CoA synthase 1 | 1.8909 | -0.61682 | 4.510E-42 | 2.300E-07 |
| HBEGF | heparin binding EGF like growth factor | 1.1016 | -1.272 | 1.270E-09 | 1.840E-11 |
| HAS3 | hyaluronan synthase 3 | 1.2446 | -1.3738 | 6.730E-06 | 1.600E-06 |
| G0S2 | G0/G1 switch 2 | 0.45425 | -0.60398 | 7.720E-06 | 1.670E-08 |
| FGFBP1 | fibroblast growth factor binding protein 1 | 0.95138 | -0.75673 | 7.900E-40 | 1.240E-25 |
| FASN | fatty acid synthase | 1.0064 | -0.40569 | 1.300E-89 | 4.960E-16 |
| FAM110C | family with sequence similarity 110 member C | 0.81763 | -1.3032 | 1.310E-04 | 2.400E-08 |
| ESM1 | endothelial cell specific molecule 1 | 1.837 | -2.7641 | 9.050E-05 | 5.320E-07 |
| ENO1 | enolase 1 | 0.2047 | -0.26034 | 1.308E-04 | 8.880E-06 |
| CXCL1 | C-X-C motif chemokine ligand 1 | 3.3047 | -2.0834 | 1.370E-06 | 2.025E-04 |
| BIRC3 | baculoviral IAP repeat containing 3 | 1.0462 | -1.0885 | 1.531E-04 | 1.242E-04 |
| AREG | amphiregulin | 0.31693 | -0.53463 | 9.380E-08 | 1.170E-17 |
| AQP3 | aquaporin 3 (Gill blood group) | 0.60868 | -0.79588 | 8.270E-05 | 1.020E-06 |
| ANXA2 | annexin A2 | 0.17626 | -0.26389 | 5.180E-06 | 2.100E-10 |
| ACTG1 | actin gamma 1 | 0.71558 | -0.48989 | 6.200E-33 | 1.840E-15 |
| ACTB | actin beta | 0.26656 | -0.24413 | 1.340E-14 | 4.560E-10 |
